# Comparison of Intaglio Surface Trueness of Interim Dental Crowns Fabricated with SLA 3D Printing, DLP 3D Printing, and Milling Technologies

**DOI:** 10.3390/healthcare9080983

**Published:** 2021-08-03

**Authors:** Keunbada Son, Jung-Ho Lee, Kyu-Bok Lee

**Affiliations:** 1 Department of Dental Science, Graduate School, Kyungpook National University, Daegu 41940, Korea; sonkeunbada@gmail.com; 2 Advanced Dental Device Development Institute, Kyungpook National University, Daegu 41940, Korea; 3 SAESHIN, 52, Secheon-ro 1-gil, Dasa-eup, Dalseong-gun, Daegu 42941, Korea; president@saeshin.com; 4 Department of Prosthodontics, School of Dentistry, Kyungpook National University, Daegu 41940, Korea

**Keywords:** trueness, 3D printing, milling, interim dental crown, digital dentistry, dental device

## Abstract

This study aimed to evaluate the intaglio surface trueness of interim dental crowns fabricated with three 3-dimensional (3D) printing and milling technologies. Dental crown was designated and assigned as a computer-aided design (CAD) reference model (CRM). Interim dental crowns were fabricated based on CRM using two types of 3D printer technologies (stereolithography apparatus and digital light processing) and one type of milling machine (*n* = 15 per technology). The fabricated interim dental crowns were obtained via 3D modeling of the intaglio surface using a laboratory scanner and designated as CAD test models (CTMs). The alignment and 3D comparison of CRM and CTM were performed based on the intaglio surface using a 3D inspection software program (Geomagic Control X). Statistical analysis was validated using one-way analysis of variance and Tukey HSD test (α = 0.05). There were significant differences in intaglio surface trueness between the three different fabrication technologies, and high trueness values were observed in the milling group (*p* < 0.05). In the milling group, there was a significant difference in trueness according to the location of the intaglio surface (*p* < 0.001). In the manufacturing process of interim dental crowns, 3D printing technologies showed superior and uniform manufacturing accuracy than milling technology.

## 1. Introduction

The introduction of dental computer-aided design and computer-aided manufacturing (CAD/CAM) systems in dental clinics is rapidly increasing [1,2,3]. Errors in operator experience and materials have been reduced due to the dental CAD/CAM system compared with the conventional methods [4,5]. Moreover, the CAD/CAM method is superior to the conventional method in terms of production time efficiency [6]. The CAD/CAM process manufactures dental prostheses in the order of scanning, CAD, and CAM processes [7,8]. The steps of CAD/CAM workflow are as follows: acquire a virtual work model using a 3-dimensional (3D) scanner and produce a working cast using a 3D printer, milling machine, or design a prosthesis in CAD software without a model and then use 3D printing and milling technologies to fabricate dental prostheses [9,10].

The manufacturing industry verifies that manufactured products are accurately manufactured [11,12,13]. Compared with visual inspection, this can save time, and the use of a 3D scanner makes accurate and quantitative analysis possible [14]. Because of the spread of dental CAD/CAM technology, several studies have evaluated the 3D data [15,16,17,18]. The accuracy was evaluated by measuring the distance from any reference point or shape [19,20]. Furthermore, in many previous studies, 3D analysis was performed by an overlapping CAD reference model (CRM), which is the basis of evaluation, and CAD test model (CTM), which is the subject of evaluation, and calculating the distances of the corresponding 3D modeling points [21,22,23]. The alignment process, overlapping with CRM on software, is an important point in the 3D analysis, and the overlapping process is generally studied via best fit alignment [24].

In the dental CAD/CAM system, CAM can be largely divided into milling and additive technologies, and 3D printing, an additive technology, is widely used for manufacture of dental interim prosthesis [25,26,27,28]. Srinivasan et al. [29] and Kalberer et al. [30] evaluated the 3D trueness to verify the volume change of the intaglio surface of the fabricated dental prosthesis. Jang et al. [31] has reported that the intaglio surface trueness of dental prostheses can affect marginal and internal fit [31]. Additionally, previous studies reported that, considering the cement space of dental prostheses, intaglio surface trueness of <100 µm was considered as a clinically applicable range [32,33]. Therefore, evaluation of the intaglio surface trueness according to various CAM technologies is still necessary for application to dental clinical practice.

Various 3D printer technologies are being applied for the fabrication of dental prostheses [25,26,27,28]. In the fabrication of dental prostheses using 3D printing with photosensitive resin, stereolithography apparatus (SLA), and digital light processing (DLP) technologies are popularly used [25,26,27,28]. The DLP 3D printer is a technology that uses a light projector to project an image to polymerize photosensitive resin [28]. The SLA 3D printer is a technology that performs layer-by-layer polymerization using ultraviolet laser to polymerize photosensitive resin [27]. Previous studies evaluated the trueness of dental prostheses using SLA and DLP techniques [25,26,27,28], but studies evaluating both SLA and DLP technologies are still lacking. Also, studies evaluating trueness according to specific areas of intaglio surface of interim crowns are still lacking, except for the present study.

Thus, this study aimed to evaluate the intaglio surface trueness of interim dental crowns manufactured with two types of 3D printer technologies (SLA and DLP) and one type of milling machine. The null hypothesis of this study was that there is no difference in the intaglio surface trueness of interim dental crowns manufactured with three types of CAM technologies.

## 2. Materials and Methods

**Figure 1:**
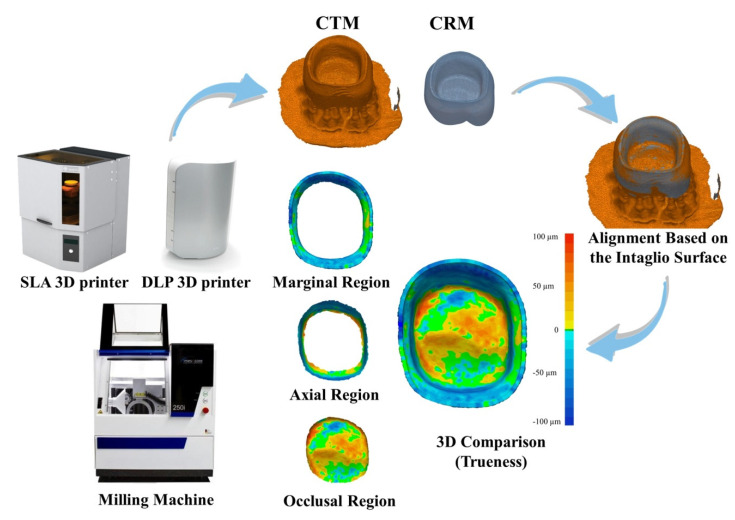
Procedure for intaglio surface trueness of interim crowns fabricated with SLA 3D printing, DLP 3D printing, and milling technologies.

A maxillary typodont model (D85DP-500B.1; Nissin dental, Kyoto, Japan) was used for the fabrication of resin abutment. The abutment of maxillary right first molar was prepared with an occlusal reduction of 1.5 mm, an axial reduction of 1.2 mm, a finish line design of the chamfer, and a convergence angle of 6°. The abutment was prepared using diamond bur (852.FG.014; Jota AG, Rüthi, SG, Switzerland) with a diameter of 1.4 mm round end taper shape, and medium roughness. A dental CAD software program (3Shape Dental System, version 17.3.0, 3Shape, Copenhagen, Denmark) was used to design a virtual crown with cement space of 80 µm based on the abutment scanned using a desktop scanner (E1, 3Shape, Copenhagen, Denmark) and acquired virtual model was designated as CRM (Figure 1).

Based on CRM, interim crowns were fabricated through the three manufacturing technologies (*n* = 15 per technology). For 3D printing technology, SLA (ZENITH U, Dentis, Daegu, Korea) with photopolymer resin for interim crown (ZMD-1000B; Dentis, Daegu, Korea) and DLP (RAYDENT Studio, Ray, Seoul, Korea) with photopolymer resin for interim crown (RAYDENT C&B; Ray, Seoul, Korea) were used (Figure 1). The 3D printing conditions were the same for both SLA and DLP, and CRM was printed under the condition of a 180° building angle with the occlusal surface facing the platform and a layer thickness of 25 µm. The manufacturer did not provide any information about the value or compensation for shrinkage that occurs during polymerization of the photopolymer resin. The interim crowns were fabricated with milling technology using a milling machine (CORITEC 250i, imes-icore GmbH, Eiterfeld, Germany). The milling rotary instruments were set to the smallest size of 0.6 mm, and wet processing was performed with prefabricated resin block (PMMA DISK; Yamahachi dental mpg, Aichi Pref, Japan). The tool path was automatically set using standard CAM software programs (iCAM V4.6; imes-icore GmbH, Eiterfeld, Germany), and the milling process was performed under the following conditions (machine configuration: five axis; milling strategy: one spindle using different instruments in z-level; diameters rotary instruments (mm): 2.5, 1.0, 0.6). The fabricated interim crowns were washed to remove all residual resin following the manufacturer’s recommendations. After interim crowns were fabricated, each interim crown was rinsed with 95% isopropyl alcohol for 5 min using an ultrasonic cleaner, followed by post-polymerization using a curing unit (CUREDEN; Kwang Myung DAICOM, Seoul, Korea) for 15 min [28]. A desktop scanner (E1, 3Shape, Copenhagen, Denmark) was used to scan the fabricated interim crowns under high-precision scan mode by designating the intaglio surface using, and the scanned virtual crowns were designated as CTMs (Figure 1). The desktop scanner used in this study was calibrated before the scanning process, and according to the manufacturer, it has a scanning accuracy of less than 10 µm. The acquisition of CTMs was completed within 2 h after the second curing in consideration of the volume change according to the passage of time.

The 3D trueness analysis was performed using 3D inspection software (Geomagic Control X v2018.0.0, 3D Systems Inc., Rock Hill, SC, USA). The CRM was loaded in the 3D inspection software, and three regions were segmented to compare the 3D trueness according to the location of the intaglio surface (Figure 1). The marginal region was the region from the crown margin to 1 mm, the axial region was the region from the end of the margin region through the axial to the point where the flat surface of the occlusal region began, and the occlusal region was the region remaining from the end of the axial region (Figure 1).

After preparing CRM, CTMs were imported and initial alignment was performed. Based on the segmented intaglio surface, best fit alignment was performed, and the sampling rate was set to all point clouds (100%) of the intaglio surface (Figure 1). Analysis of 3D trueness was performed by calculating all point cloud points of the segmented intaglio surface of CRM. At this time, each corresponding data point in CRM and CTM was calculated as the root mean square (RMS) value as shown in Formula (1):

(1) $$RMS=\frac{1}{\sqrt{n}}\cdot \sqrt{\overset{n}{\underset{i=1}{\sum }}{\left(X_{1,i}-X_{2,i}\right)}^{2}}$$

For all data points, $X_{1,i}$ is the CRM, $X_{2,i}$ is the coordinate at *i* time in the CTM, and *n* is the number of all data points measured in each analysis. The RMS value shows how the shapes of different virtual models are different in 3D, and a low RMS value means a high degree of matching of the superimposed virtual models. The 3D comparison was shown as a color difference map, and a range of ±100 µm (20 color segments) and a tolerance range of ±10 µm (green) were specified (Figure 1).

To determine the sample size, an appropriate sample size was calculated as 15 using power analysis (G*Power v3.1.9.4, Heinrich-Heine-Universität, Dusseldorf, Germany) based on the results of five pilot experiments (SLA group: 24.7 ± 6.0 µm; DLP group: 30.8 ± 2.8 µm; milling group: 49.0 ± 2.1 µm; effect size [f] = 0.86; actual power = 99.94%; power = 99.9%; α = 0.05). All data analyses were performed using statistical software (IBM SPSS Statistics v23.0, IBM Corp, Armonk, NY, USA). First, the normal distribution of the data was investigated using the Shapiro–Wilk test, and the normal distribution of the obtained data was confirmed. Therefore, the differences between groups were confirmed using one-way analysis of variance (ANOVA) and analyzed using the Tukey HSD test as a post hoc test (α = 0.05). The interaction effect between the evaluated region and the manufacturing technology was verified using two-way ANOVA (α = 0.05).

## 3. Results

There were significant differences in intaglio surface trueness in all regions among SLA, DLP, and milling groups (Table 1; *p* < 0.001). Except for the occlusal region, there was no significant difference between SLA and DLP in the whole, marginal, and axial regions (Table 1; *p* > 0.05), but there was a significant difference between the milling and 3D printing group (Table 1; *p* < 0.05). SLA (23.6 ± 5.3 µm), DLP (29.0 ± 3.6 µm), and milling groups (36.9 ± 4.4 µm) showed significantly higher intaglio surface trueness in the order in the occlusal region (Table 1; *p* < 0.05). According to the results of two-way ANOVA, there was a significant interaction effect between the evaluated region and the manufacturing technology (F = 3.699; *p* = 0.002).

There was no significant difference between SLA (*p* = 0.219) and DLP groups according to the locations of the intaglio surface (*p* = 0.122) (Table 2). However, the milling group showed a significant difference according to the locations of the intaglio surface and showed lower intaglio surface trueness in the occlusal region than that in the marginal and axial regions (Table 2; *p* < 0.001).

**Figure 2:**
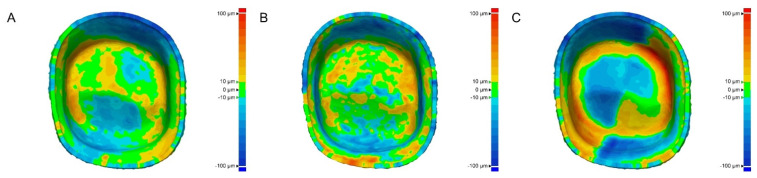
Schematic of color difference map of intaglio surface trueness of interim crowns. (**A**) SLA. (**B**) DLP. (**C**) Milling.

In the color difference map, SLA and DLP did not have a specific color distribution in any region, but in the milling group, there was a high amount of trueness (red color) in the axial and angular regions of the intaglio surface (Figure 2).

## 4. Discussion

In this study, three types of fabrication technologies were used to fabricate interim dental crowns and the intaglio surface trueness was evaluated. The null hypothesis of this study was rejected because there was a significant difference in the intaglio surface trueness of interim dental crowns manufactured with the three types of CAM technologies (*p* < 0.05). Previous studies have evaluated the intaglio surface trueness of dental crowns [29,30]. In a previous study, the 3D trueness of zirconia crowns fabricated using 3D printing was evaluated to investigate the potential application of 3D printing technology in a study on dental ceramic restorations [34]. In another study, the 3D printing group (38 ± 12 µm) showed significantly better intaglio surface trueness than the milling group (43 ± 12 µm) (*p* < 0.001), and the 3D printing group showed the same results as those reported in this study that showed superior results in the 3D printing group (Table 1) [25]. Another previous study evaluated the trueness of zirconia crowns fabricated by printing with 3D gel deposition technology [26]. The results of this study (Table 1) and the study by Wang et al. [25] showed that the 3D printing group showed significantly better intaglio surface trueness than the milling group. In light of the results of previous studies and this study, 3D printing technology is considered to have sufficient manufacturing accuracy for clinical application.

The intaglio surface trueness of dental prostheses fabricated with various materials and methods have been reported in many previous studies [25,26,27,28,29,30,34]. A previous study has reported the intaglio surface trueness (28.5 ± 6.0 µm) of interim crowns fabricated by printing with SLA technology [27]. These results showed similar trueness to that of this study (SLA: 25.7 ± 5.1 µm) (Table 1). Another previous study has reported the intaglio surface trueness (24.91 ± 3.62 µm) of interim crowns fabricated by printing with DLP technology [28]. These results showed trueness similar to that observed in this study (DLP: 29.5 ± 3.3 µm) (Table 1). Furthermore, another previous study has reported the intaglio surface trueness (42.9 ± 4.4 µm) of crowns fabricated with milling technology [7]. These results showed trueness similar to that observed in this study (milling: 44.8 ± 5.5 µm) (Table 1). Therefore, despite the differences in in vitro experimental conditions, the results of previous studies and this study showed similar trends. Additionally, previous clinical studies evaluated the intaglio surface trueness (43.8 ± 11.7 µm) of ceramic crowns fabricated with milling technology [8] and showed trueness similar to that observed in this study (milling: 44.8 ± 5.5 µm). In previous studies, the intaglio surface trueness of <100 µm was recommended based on the cement space of the fixed dental prosthesis as an error may occur in the manufacturing process [32,33]. Therefore, in terms of intaglio surface trueness, interim crowns evaluated in this study can be considered appropriate for clinical use, and 3D printing can be considered to have superior intaglio surface trueness than milling technology.

The trueness evaluation of interim crowns performed in previous studies compared the results of milling technology and 3D printing technology [27,28]. The present study compared the results of SLA and DLP of 3D printing technology, including the comparison of milling technology and 3D printing technology, and reported the similar trueness of interim crowns between SLA and DLP (Table 1). The results of this study showed that the interim crowns fabricated with 3D printing technology showed the same results regardless of the evaluated region, but the milling technology showed different results of trueness according to the locations of the intaglio surface (Table 2). Furthermore, a previous study reported that milling technology could have different trueness according to the region of the intaglio surface of the crown [7]. In this study, Figure 2C shows an error in the angle region between the axial and occlusal regions, and these results are similar to those reported in previous studies [6,7]. During the milling process, this machining error was reported as a machining limitation due to the size of the diameter of the burr used and may appear when machining angle region of the intaglio surface [6,7]. Milling technology reported that the number of burrs affects the accuracy, and trueness is better when using many burs [6]. Using a smaller diameter bur increases manufacturing time due to increased tool path, but may yield better trueness results because a wider range of bur diameters is created [6]. For this reason, using a smaller diameter burr allows for more accurate milling of the angle region of the intaglio surface [7]. Therefore, the error in the angle region between the axial and occlusal regions in crowns must be considered during milling. Additional studies through trueness evaluation using burs of various diameters are needed.

SLA and DLP technologies are one of the most used additive manufacturing processes in dentistry, offering the highest accuracy and resolution of any printing technology, superior detail and smooth surface finish [35]. It is then built through the deposition of successive layers of a photosensitive material that polymerizes easily [35]. SLA is the first rapid prototyping technology with a reliable printing process [27]. So far, SLA is the only photocurable 3D printing technology that can print large-format models, but SLA has a low printing rate due to the curing rate caused by the movement of the laser beam, so the larger the model, the slower the printing speed [36]. However, DLP 3D printing uses a digital projector screen to flash an image in layers across the entire platform, curing all points at the same time, so it has the advantages of high precision and fast manufacturing times [37]. However, only small sized objects can be printed because the projection size is limited to ensure high precision. Volume shrinkage is also reported as a disadvantage of photocurable 3D printing [28]. Milling technology, a subtractive manufacturing process, reproduces shapes by cutting using milling equipment and burs [30]. Therefore, the material loss is relatively large, and the reproducibility is limited by the diameter of the burr [30].

This study has some limitations. First, the effect of intaglio surface trueness on the actual clinical environment should be investigated via additional clinical studies. Second, 3D printers and milling equipment from more diverse manufacturers should be used to confirm additional results. Third, the trueness of external surfaces including intaglio surfaces should be evaluated via additional studies.

## 5. Conclusions

Based on the findings of this in vitro study, the following conclusions were drawn. The 3D printing and milling technologies used in this study showed clinically acceptable intaglio surface trueness (<100 µm) of interim crowns. The milling technology showed inferior trueness in the reproduction of angle region than occlusal region. However, interim crowns fabricated with 3D printing technologies (SLA and DLP) can reproduce more uniform and superior intaglio surface trueness than milling technology.

## Figures and Tables

**Table 1:** Comparison of intaglio surface trueness (µm) of interim crowns fabricated with SLA 3D printing, DLP 3D printing, and milling technologies.

Evaluated Region	Manufacturing	Mean	SD	95% Confidence Interval (CI)		Minimum	Maximum	F	*p*
				Lower	Upper				
Whole region	SLA	25.7 ^A^	5.1	22.8	28.6	18	34.2	66.684	<0.001 *
	DLP	29.5 ^A^	3.3	27.6	31.3	24.4	36.8		
	Milling	44.8 ^B^	5.5	41.7	47.9	33	53.2		
Marginal region	SLA	26.7 ^A^	4.4	24.2	29.2	20.2	34	45.267	<0.001 *
	DLP	27.0 ^A^	4.7	24.3	29.6	20.4	37.3		
	Milling	45.2 ^B^	8.2	40.6	49.8	35.8	59.4		
Axial region	SLA	27.6 ^A^	6.5	24	31.3	17.6	40.9	47.674	<0.001 *
	DLP	30.9 ^A^	5.6	27.8	34	23.6	40.6		
	Milling	50.5 ^B^	8.3	45.9	55.2	34	63.1		
Occlusal region	SLA	23.6 ^A^	5.3	20.6	26.5	17	33.4	32.288	<0.001 *
	DLP	29.0 ^B^	3.6	26.9	31	24.3	35.5		
	Milling	36.9 ^C^	4.4	34.4	39.3	29.1	45.5		

* Significant difference by one-way ANOVA; *p* < 0.05. Different letters indicate significant differences among the three methods by the Tukey HSD test (*p* < 0.05).

**Table 2:** Comparison of intaglio surface trueness (µm) of interim crowns according to the evaluated regions.

Evaluated Region	SLA	DLP	Milling
Whole region	25.7 ± 5.1	29.5 ± 3.3	44.8 ± 5.5 ^A^
Marginal region	26.7 ± 4.4	27.0 ± 4.4	45.2 ± 8.2 ^A^
Axial region	27.6 ± 6.5	30.9 ± 5.6	50.5 ± 8.3 ^A^
Occlusal region	23.6 ± 5.3	29.0 ± 3.6	36.9 ± 4.4 ^B^
F	1.52	2.016	10.025
*p*	0.219	0.122	<0.001 *

* Significant difference by one-way ANOVA; *p* < 0.05. Different letters (A and B) indicate significant differences among the evaluated regions by the Tukey HSD test (*p* < 0.05).

## Data Availability

Data are included within the article.
